# Hyaluronan: Metabolism and Function

**DOI:** 10.3390/biom10111525

**Published:** 2020-11-07

**Authors:** Takashi Kobayashi, Theerawut Chanmee, Naoki Itano

**Affiliations:** 1 Institute for Molecular Science of Medicine, Aichi Medical University, Nagakute, Aichi 480-1195, Japan; t-kobayashi@aichi-med-u.ac.jp; 2 Department of Clinical Chemistry, Faculty of Medical Technology, Mahidol University, Phutthamonthon, Nakhon Pathom 73170, Thailand; theerawut.cha@mahidol.ac.th; 3 Department of Molecular Biosciences, Faculty of Life Sciences, Kyoto Sangyo University, Kita-ku, Kyoto 603-8555, Japan

**Keywords:** hyaluronan, metabolism, biosynthesis, degradation, extracellular matrix, cancer

## Abstract

As a major polysaccharide component of the extracellular matrix, hyaluronan plays essential roles in the organization of tissue architecture and the regulation of cellular functions, such as cell proliferation and migration, through interactions with cell-surface receptors and binding molecules. Metabolic pathways for biosynthesis and degradation tightly control the turnover rate, concentration, and molecular size of hyaluronan in tissues. Despite the relatively simple chemical composition of this polysaccharide, its wide range of molecular weights mediate diverse functions that depend on molecular size and tissue concentration. Genetic engineering and pharmacological approaches have demonstrated close associations between hyaluronan metabolism and functions in many physiological and pathological events, including morphogenesis, wound healing, and inflammation. Moreover, emerging evidence has suggested that the accumulation of hyaluronan extracellular matrix and fragments due to the altered expression of hyaluronan synthases and hyaluronidases potentiates cancer development and progression by remodeling the tumor microenvironment. In addition to the well-known functions exerted by extracellular hyaluronan, recent metabolomic approaches have also revealed that its synthesis can regulate cellular functions via the reprogramming of cellular metabolism. This review highlights the current advances in knowledge on the biosynthesis and catabolism of hyaluronan and describes the diverse functions associated with hyaluronan metabolism.

## 1. Introduction

**Figure 1:**
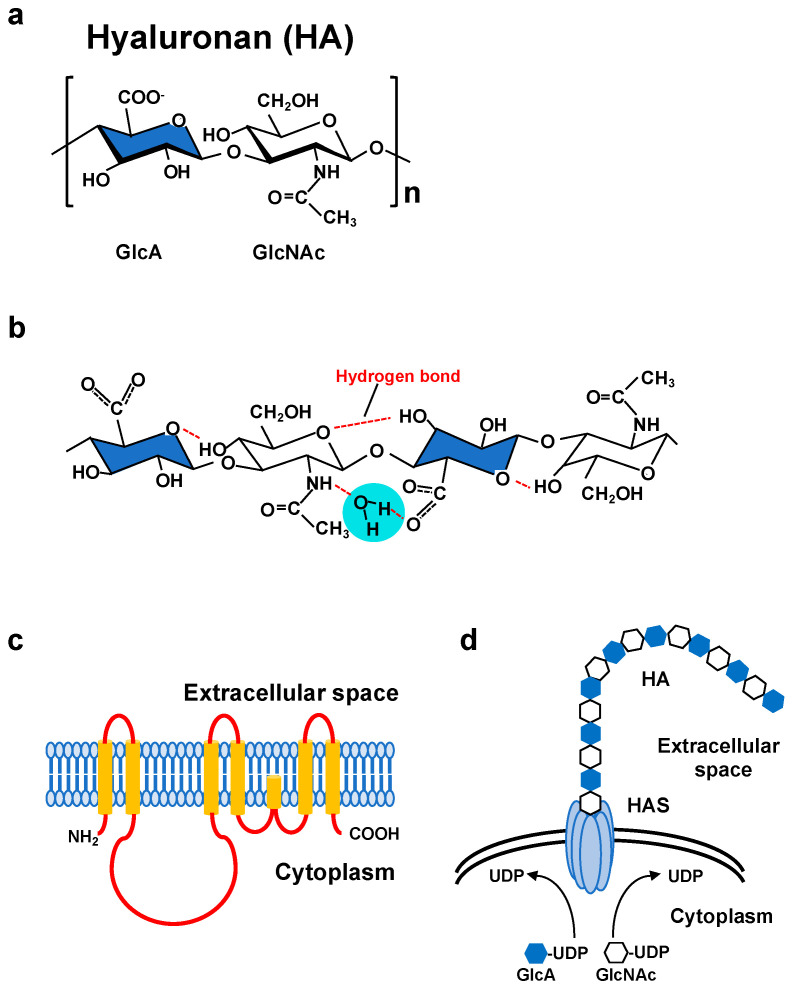
(**a**) Molecular structure of a HA disaccharide unit. HA is a negatively charged polysaccharide composed of repeating disaccharide units of glucuronic acid (GlcA; blue) and *N*-acetylglucosamine (GlcNAc). (**b**) Secondary structure of a HA tetrasaccharide with water. Hydrogen bonds are represented by red dashed lines. (**c**) Predicted structure of mammalian HAS. HAS enzymes contain multiple membrane-spanning regions at both the amino and carboxyl terminus and catalytic sites at the central part of the molecule. (**d**) Schematic illustration of HA synthesis and secretion. HAS enzymes catalyze the alternative addition of UDP-GlcA and UDP-GlcNAc to the nascent HA chain and extrude it through the plasma membrane.

Hyaluronan (HA) comprises a major component of the extracellular matrix (ECM) in vertebrate connective tissues and is abundant in the cartilage, skin, brain, vitreous body, umbilical cord, and synovial fluid. Since HA was first discovered in bovine vitreous as a novel glycosaminoglycan [1], its structure, physical properties, physiological activity, and metabolism have been studied for almost a century. The HA polysaccharide is a non-sulfated linear glycosaminoglycan composed of repeating disaccharide units of [3)-β-d-*N*-acetylglucosamine (GlcNAc)-β(1,4)-d-glucuronic acid (GlcA)-β(1] (Figure 1a) [2]. The concentration and size distribution of HA vary with tissue type, age, and disease severity [3,4]. With a broad molecular weight range, HA has multiple physical and physiological properties that depend on its molecular weight and concentration, both of which are regulated by the balance between HA biosynthesis and degradation [5]. In vertebrates, the dynamic metabolism of HA is tightly controlled by three synthases and several hyaluronidases [5]. The three isoforms of HA synthases (HAS1, HAS2, and HAS3), each with different characteristics and regulatory systems, control HA biosynthesis at multiple stages [6]. Among the HYAL family members of hyaluronidases, HYAL1 and HYAL2 are widely expressed in mammalian tissues and are thought to be major contributors to HA catabolism [7]. More recently, cell migration inducing protein (CEMIP)/KIAA1199 and transmembrane protein 2 (TMEM2) have been identified as novel molecules involved in extracellular HA degradation [8,9].

**Figure 2:**
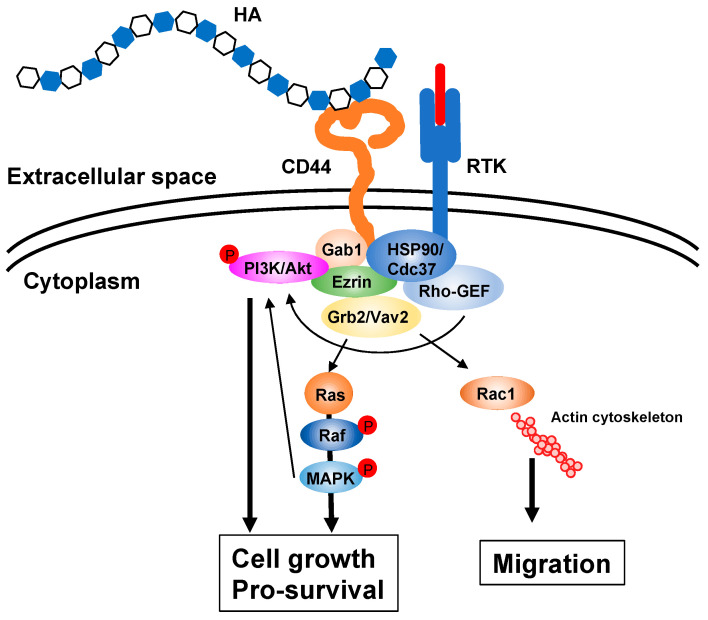
HA signaling cascades. HA ECM activates intracellular signaling cascades via receptors such as CD44 and upregulates several genes related to cell proliferation and survival. The interaction of HA with its receptor also induces actin cytoskeleton rearrangement, leading to active cell migration. Gab1; Grb2-associated-binding protein 1, GEF; guanine nucleotide exchange factor, Grb2; growth factor receptor-bound protein 2, HSP90; heat shock protein 90, MAPK; mitogen-activated protein kinase, PI3K; phosphoinositide 3-kinase, RTK; receptor tyrosine kinase.

HA is a biopolymer with excellent water retention ability and can form a meshwork structure. As high molecular weight (HMW) HA is stabilized by intermolecular and intramolecular interactions with hydrogen and hydrophobic bonds in an aqueous solution, highly concentrated solutions exhibit considerable viscoelasticity (Figure 1b) [10]. Due to its low diffusivity, HMW HA forms a pericellular ECM around its producing cells. The composition and function of the HA ECM are multiply regulated by association states and combinations with specific binding molecules [11,12,13]. HA not only functions as a structural framework, but also activates intracellular signal transduction by interacting with cell surface receptors for the regulation of such dynamic cell behaviors as cell proliferation, adhesion and migration, all of which are suggested to be involved in morphogenesis and wound healing [14]. The HA receptor CD44 participates in many physiological and pathological processes by interacting with HA and activating key signaling cascades [15] (Figure 2). Such interactions initiate the expression of genes related to cell growth and survival and induce cytoskeletal rearrangement and membrane ruffling, leading to active cell migration. On the other hand, HA fragments degraded by the action of hyaluronidases diffuse throughout tissues and bind to HA receptors on peripheral cells to act as intercellular signals [14]. Thus, HA has a variety of functions that cannot be easily imagined from its simple structure. These functions are controlled by modulating concentration, sugar chain length, turnover rate, and other features of HA as well as by HA association states with binding molecules.

Abnormalities in HA metabolism have been associated with inflammatory disease severity and cancer aggressiveness [16]. In rapidly progressing malignancies, HA biosynthesis and degradation are significantly enhanced as compared with normal tissues, which may increase HA matrix formation and the production of HMW HA and its fragments [17]. Thus, cancer cells are exposed to a unique microenvironment where both HA matrix and fragments co-exist. With its excellent water retention, HMW HA forms a pericellular ECM favorable for cancer cell proliferation and migration. On the other hand, the fragments generated by HA degradation promote the growth of endothelial cells and thereby induce angiogenesis [18]. Furthermore, HA fragments possess significant immunomodulatory activity, resulting in diminished tumor immune surveillance [19]. Therefore, tumor microenvironment (TME) remodeling by the excessive production and degradation of HA contributes to the survival and malignant progression of cancer cells. Recent metabolomic approaches have also clarified that HA regulates cellular functions via the reprogramming of cellular metabolism coupled with its production [20,21]. We earlier discovered that HA synthesis promoted cancer stem cell (CSC)-like properties through the metabolic reprogramming of glycolysis and the hexosamine biosynthetic pathway (HBP) [20]. The above findings strongly suggest that HA not only has canonical functions as an ECM component, but also acts as a key regulator of cellular metabolism. In this review, we first describe the current advances in knowledge on HA metabolism. We subsequently discuss the close associations between HA metabolism and functions, referring to recent discoveries in metabolic reprogramming coupled with HA production.

## 2. HA Biosynthesis

HA is a non-sulfated glycosaminoglycan in which GlcNAc and GlcA are linked together by alternating β-1,3 and β-1,4 linkages (Figure 1a). The biosynthesis of HA greatly differs from that of other glycosaminoglycans [2]. Since the cloning of genes encoding HA synthases from Streptococci and mammalian cells, our understanding of the HA biosynthetic mechanism has progressed considerably [5,22,23]. In vertebrates, three HAS family members whose amino acid sequences are conserved across species have been identified. Primary structure analysis has revealed that all HAS enzymes contain multiple clusters of hydrophobic amino acids at both the amino and carboxyl terminus, indicating that they are inserted into the lipid bilayer (Figure 1c). The central part of the HAS molecule is composed of relatively hydrophilic amino acids and possesses the catalytic sites necessary for transferring the UDP-GlcNAc and UDP-GlcA substrates (Figure 1d).

Biochemical analyses have revealed that each HAS isoform differs in terms of activity, product elongation rate, and stability [6]. The length of HA synthesized in vitro and in vivo by each enzyme varies as well. The reason for plural HAS isoforms is presumed to be that multiple enzymes possessing different enzymatic properties are functionally complementary to each other. In fact, HA biosynthesis is regulated at several stages by regulating the expression of the three HAS isoforms. Among the mammalian HAS isoforms, the activity and turnover of HAS2 have been demonstrated to be posttranslationally controlled by phosphorylation, *O*-GlcNAcylation, ubiquitination, and dimerization [24,25,26,27]. Moreover, the natural antisense transcript HAS2 antisense RNA 1 regulates the stabilization of HAS2 mRNA [28]. HA synthesis is therefore strictly and multiply regulated by one or a combination of the above regulatory mechanisms.

**Figure 3:**
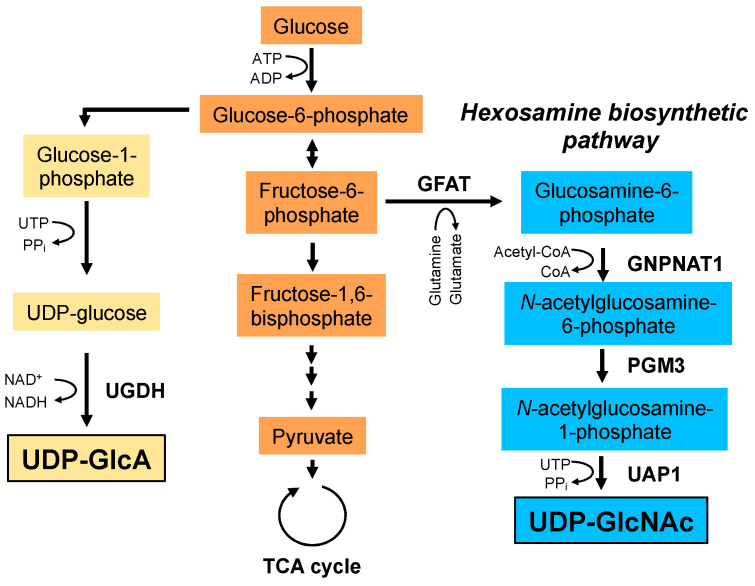
Biosynthetic pathways of UDP-GlcA and UDP-GlcNAc. UDP-GlcA is synthesized from glucose-6-phosphate. Glucose-6-phosphate is converted to glucose-1-phosphate and UDP-glucose. Finally, UDP-GlcA is synthesized from UDP-glucose by UDP-glucose dehydrogenase (UGDH). UDP-GlcNAc is synthesized via the hexosamine biosynthetic pathway. Glutamine: fructose-6-phosphate amidotransferase (GFAT) utilizes glutamine as an amine donor to generate glucosamine-6-phosphate. In the next step, acetyl-CoA is transferred to glucosamine-6-phosphate by glucosamine-phosphate *N*-acetyltransferase 1 (GNPNAT1). The resulting *N*-acetylglucosamine-6-phosphate is then converted to *N*-acetylglucosamine-1-phosphate by phosphoglucomutase 3 (PGM3). In the final step, uridine-5‘-triphosphate (UTP) is transferred to *N*-acetylglucosamine-1-phosphate by UDP-GlcNAc pyrophosphorylase (UAP1) to form the end product, UDP-GlcNAc. PPi; pyrophosphate.

HA synthesis is influenced by the cellular availability of UDP-GlcNAc and UDP-GlcA nucleotide sugar donors (Figure 3). For example, HA synthesis inhibitor 4-methylumbelliferone (4-MU; 7-hydroxy-4-methylcoumarin) inhibits HA synthesis by depleting cytoplasmic UDP-GlcA [29]. Similarly, depletion of cellular UDP-GlcNAc by mannose treatment reduces HA synthesis in epidermal keratinocytes [30]. UDP-GlcA is a key metabolite in glycosaminoglycan biosynthesis and is synthesized from glucose-6-phosphate, an intermediate metabolite of glycolysis, through the synthesis of glucose-1-phosphate and UDP-glucose. UDP-GlcNAc is synthesized via the HBP, a branch of the main glycolytic pathway and is utilized as a donor substrate for protein glycosylation and *O*-GlcNAcylation. HA synthesis consumes large quantities of its donor substrates, thereby linking HA synthesis to glucose metabolism. As described later, recent mass spectrometry profiling has revealed that HA overproduction accelerated the flux of glucose carbon through the HBP (i.e., HBP flux) and shifted cellular metabolism towards glycolysis in breast cancer cells [20]. Moreover, HAS2 overexpression shifted the metabolic profile in chondrocytes from glycolysis to mitochondrial respiration [21] and the inhibition of HA synthesis by 4-MU treatment shifted glucose flux to glycolysis in brown adipose tissue (BAT) [31]. These contradictory consequences of metabolic shifts might be due to the context of different cell lines. In any case, they provide supportive evidence for a novel mechanism by which the consumption of nucleotide sugar donors by HA biosynthesis affects glucose metabolism.

## 3. HA Catabolism

**Figure 4:**
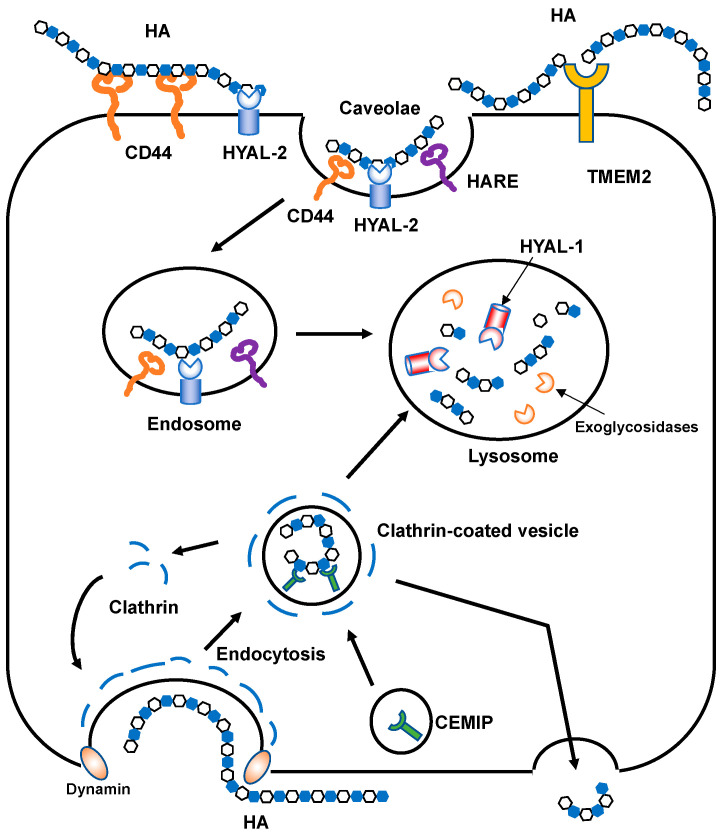
Schematic illustration of HA degradation mechanisms. In HYAL-mediated HA degradation (top), HMW HA is tethered to the cell surface by CD44 and GPI-anchored HYAL2 into caveolin-rich lipid rafts and then cleaved into approximately 2 × 10^4^ Da fragments. The HA fragments are subsequently delivered to endo-lysosome compartments and degraded into smaller oligosaccharides by HYAL1 and monosaccharides by exoglycosidases. In the proposed model of CEMIP-mediated HA degradation (bottom), HMW HA is endocytosed into clathrin-coated vesicles and cleaved into lower molecular weight HA fragments by the action of CEMIP, which is localized in the peripheral vesicles of the cell. The fragmented HA is then depolymerized in endo-lysosome compartments or released extracellularly without intracytoplasmic accumulation. TMEM2 is expressed on the cell surface in a type II transmembrane topology and degrades HMW HA into approximately 5 × 10^3^ Da fragments.

HA is normally depolymerized by specific endoglycosidases called hyaluronidases or is non-specifically degraded by oxidative damage due to reactive oxygen species (ROS). Mammalian hyaluronidases hydrolyze the β-1,4-glycosidic bond between the GlcNAc and GlcA of HA. In humans, six HYAL family members have been identified and share approximately 40% identity [32]. The HYAL1, HYAL2, and HYAL3 genes are clustered on human chromosome 3p21.3, while the HYAL4, PH-20/sperm adhesion molecule 1 (SPAM1), and HYALP1 genes are located on chromosome 7p31.3 [7]. Considering their relatively high sequence homology, these two clusters may have been generated by gene duplication. HYAL1 and HYAL2 are major mammalian hyaluronidases and are broadly expressed in somatic tissues. HYAL3 is also weakly expressed in a wide range of somatic cells. However, it exhibits no detectable in vitro activity [33], and Hyal3 null mice do not accumulate HA [34]. The enzymes encoded by the latter cluster have somewhat different roles from those encoded by the former. HYAL4 has recently been reidentified as a chondroitin sulfate (CS)-specific hydrolase and does not act on HA [35]. PH-20/SPAM1 is a glycosylphosphatidylinositol (GPI)-anchored hyaluronidase in the testes that plays essential roles in fertilization [36]. PH-20 is expressed at the anterior sperm head surface and promotes sperm penetration through the cumulus cells that surround the oocyte embedded in the HA-rich ECM. HYALP1 is an active enzyme in mice but is an expressed pseudogene in humans [7]. Recently, two novel molecules involved in extracellular HA degradation have been identified in mammals. First, CEMIP, alternatively called KIAA1199 or hyaluronan binding protein involved in hyaluronan depolymerization (HYBID), is a 150 kDa protein with an N-terminal secretion signal peptide [8]. CEMIP does not display homology with other HYAL family proteins. CEMIP-mediated HA depolymerization is considered to occur via the clathrin-coated pit pathway (Figure 4). Second, TMEM2 is a type II transmembrane protein with sequence similarities to CEMIP [9]. TMEM2 cleaves HMW HA into approximately 5 × 10^3^ Da fragments in a Ca^2+^-dependent manner under neutral pH.

In the proposed model of HA catabolism, the GPI-anchored cell surface hyaluronidase HYAL2 cleaves HMW HA into approximately 2 × 10^4^ Da fragments at the cell surface with the HA receptor CD44 (Figure 4). The partially fragmented HA is internalized by binding to the HA receptors [37,38] and is then further degraded by HYAL1 and exoglycosidases in the lysosomal system. However, HYAL2 favors an acidic pH for its hyaluronidase activities [39], which are weaker than those of other HYALs [40,41]. As mentioned above, the recently identified CEMIP and TMEM2 contribute to the digestion of extracellular HMW HA into smaller fragments. Thus, fragmented HA (approximately 2 × 10^4^ Da) is likely produced by mechanisms involving HYAL2, CEMIP, and TMEM2, and in part by ROS-mediated non-specific degradation. As the fragmented HA in the ECM is usually internalized by cells through receptors, HA receptors have roles not only in intracellular signaling, but also in HA clearance. Injected HA was found to be sequestered into rabbit and rat livers in tracer experiments in the early 1980s [42,43]. Later, a protein receptor called HA receptor for endocytosis (HARE) was identified from liver sinusoidal endothelial cells [44]. HARE is a 175 kDa polypeptide generated by the proteolytic cleavage of a larger 300 kDa full-length receptor named stabilin 2, which has a link module as an HA-binding domain. HARE acts as a mediator for the rapid endocytosis of HA via clathrin-coated pit pathways [45,46].

## 4. Close Associations between HA Metabolism and Functions

### 4.1. HA Properties

The physico-chemical properties of HA are the unique features distinguishing this polysaccharide from other glycosaminoglycans. HA has remarkable hydration capacity and can swell to many times its original volume upon water absorption. HA in an aqueous solution forms a double helical structure stabilized by intermolecular hydrogen bonds between the acetamide of GlcNAc and the carboxyl group of GlcA and through interactions between hydrophobic patches (Figure 1b) [47]. In highly concentrated HA solutions, the molecules form a meshwork structure and exhibit elasticity by self-association between each other as well as by steric interactions [48]. When a continuous force is applied in a certain direction, a part of the meshwork structure aligns and exhibits viscosity while partially maintaining intermolecular interactions. Due to these physical properties, HA functions as a three-dimensional meshwork having remarkable viscoelasticity [49]. HA often organizes as a matrix meshwork structure through the electrostatic and hydrophobic interactions with its binding molecules. Within the organized structure of the HA meshwork, proteins and other macromolecules are partially excluded from the matrix region or their diffusion rates decrease due to the molecular sieving effect of the meshwork structure [50]. HA exists as a polyanion in which the carboxyl group of the GlcA residue is ionized at physiological pH [51]. In such a state, the tissue distributions of positively charged nutrients and electrolytes are affected by weak interactions with negatively charged HA. The HA receptor CD44 selectively recognizes HA of varying lengths and initiates signaling responses upon binding HA fragments of a specific size range [52]. Toll-like receptor (TLR) 2 and 4 preferentially recognize small HA fragments [53,54]. Because different sized HA molecules exhibit a wide variety of conformations, size-specific receptor recognition can be attributed to the multitude of HA conformations [55].

The properties of HA are strongly influenced by its sugar chain length and concentration. As such, changes in HA metabolism greatly affect tissue morphogenesis and homeostasis by dynamically modulating the functions of HA as a viscoelastic fluid, molecular sieve, space filler, cellular scaffold, hydrophilic reservoir or signaling molecule. Genetic engineering and pharmacological approaches to artificially alter HA metabolism have provided new insights into HA functions in morphogenesis and diseases. The close associations between HA metabolism and several functions are exemplified below.

### 4.2. Importance of HA Metabolism in Morphogenesis and Wound Healing

Due to its physico-chemical properties, HA either alone or through interactions with associated molecules, acts as a scaffold for assembling a pericellular matrix, offers a favorable microenvironment for cell proliferation and migration, transmits signals, and generates mechanical forces by tissue swelling during morphogenesis and wound healing [56].

#### 4.2.1. Cardiovascular Development

Crucial roles of HA synthesis and degradation in cardiovascular development have been demonstrated in genetically engineered mice. Has2 null mouse embryos exhibited clear growth retardation by roughly E9.0 along with cardiovascular defects [57,58]. Interestingly, the embryos also failed to undergo endothelial-to-mesenchymal transition in atrioventricular canal cushions. Via several lines of evidence, it was suggested that HA regulated atrioventricular canal differentiation through activation of the ErbB-Ras signaling pathway. On the other hand, Hyal2 knockout mice displayed high preweaning lethality, with surviving animals exhibiting atrial enlargement, cor triatriatum, and valve thickening [59,60]. In the knockout mice, HA accumulated in the ECM of valves and the interstitial ECM of atrial cardiomyocytes, which disorganized the ECM and expanded the spongiosa layer. HA accumulation by the absence of Hyal2 also promoted endothelial-to-mesenchymal transition and mesenchymal cell proliferation, resulting in excess mesenchymal cells and causing such heart structure abnormalities as thickened valves and atrial masses [61].

#### 4.2.2. Skeletal Development

HA has pivotal functions in skeletal development as a major component of the ECM. Matsumoto et al. specifically deleted the Has2 gene in developing limbs using conditional Has2 knockout mice and demonstrated a key role of HA in the growth of limb skeletal elements, digit patterning, chondrocyte maturation, and joint formation [62]. In the Has2 mutant limbs, the skeletal elements were severely shortened and the proximal phalanges were duplicated. Furthermore, the growth plates of skeletal elements were severely disorganized and the number of hypertrophic chondrocytes was strikingly reduced in the mutants. The growth plate and hypertrophic chondrocyte aberrations were coincident with a decrease in aggrecan deposition in the ECM. This was further confirmed by showing that Has2 knockout chondrocytes were unable to construct a pericellular HA matrix using the CRISPR/Cas9 gene editing approach [63]. Similar cartilage defects were observed in hyaluronidase knockout mice; Hyal2 knockout mice exhibited localized congenital defects in frontonasal and vertebral bone formation [41]. Cemip knockout mice also developed long bones that were shorter than those of wild type animals [64]. Such studies confirm that HA metabolic regulation is essential in skeletal development.

#### 4.2.3. Intestinal Development

The gut is an organ that develops with stereotypical left-right asymmetry. Sivakumar et al. demonstrated that covalent binding of HA and inter-α-trypsin inhibitor (IαI) mediated by tumor necrosis factor-stimulated gene-6 (TSG-6) promoted the accumulation of HA ECM on the right side of the dorsal mesentery (DM) [65]. Due to the large hydrodynamic volume of HA, HA matrix accumulation was considered to drive leftward gut tilting by expanding the right side of the DM. To test for the requirement of HA synthesis and accumulation in DM expansion, embryos were treated with resin beads soaked in 4-methylumbelliferone-β-D-xyloside (MU-Xyl), an inhibitor of HA synthesis, or electroporated with the Hyal2 gene to selectively degrade extracellular HA. Both MU-Xyl-treated and Hyal2-electroporated embryos had significantly reduced levels of HA in the right DM with a corresponding marked reduction in ECM expansion, suggesting that HA-mediated hydration contributed to the expansion of DM on the right side.

#### 4.2.4. Wound Healing

Wound healing is a multi-step process that involves scab formation, inflammation, granulation tissue formation, and scar formation. HMW HA accumulates at the wound site in the early stages of wound repair and becomes progressively fragmented throughout stage progression [66]. The space generated by the HA ECM facilitates the infiltration of inflammatory cells and fibroblasts to the wound site. Fibroblasts are known as a major source of hyaluronidases. At the later stages of wound repair, the generated HA fragments stimulate blood vessel formation owing to their pro-angiogenic activity. Mack et al. investigated the role of HA in wound repair and found that wound closure was significantly accelerated in Has1/Has3 double-knockout mice over wild-type animals [67]. The Has1/3 null skin showed a decrease in epidermal HA at the wound edge, a noteworthy increase in neutrophil efflux from cutaneous blood vessels, and an earlier onset of myofibroblast differentiation. Interestingly, they observed an increase in dermal Has2 expression in the Has1/3 null skin after wounding. Thus, the faster wound closure in Has1/3 null mice might have been due to compensatory upregulation of the Has2 gene. Similarly, topical application of testicular hyaluronidase accelerated wound closure in full-thickness excisional wounds in rats [68]. Buhren et al. also observed that hyaluronidase treatment promoted wound closure with a significant upregulation of HAS gene expression in an in vitro model using normal human dermal fibroblasts [69]. The above findings indicate that hyaluronidase exerts multiple effects on HA metabolism and functions in dermal fibroblasts by affecting HAS gene expression as well as by depolymerizing HMW HA.

### 4.3. Altered HA Metabolism in Inflammatory Diseases

HA deposition and degradation play essential roles in regulating inflammatory responses. Expression of the genes related to HA biosynthesis and degradation are upregulated in response to inflammatory cytokines, resulting in increased HA turnover during inflammatory responses. Fragments generated by the hyaluronidase-catalyzed digestion of HMW HA function as pro-inflammatory damage-associated molecular patterns and stimulate inflammation via TLR2 and/or TLR4 in immune cells. However, a recent study demonstrated that endotoxin-free HA fragments failed to stimulate macrophages or dendritic cells to produce inflammatory cytokines and questioned whether HA fragments directly promoted inflammation [70]. The formation of HA cable-like structures has been implicated in the pathogenesis of several inflammatory diseases [71]. These structures, whose formation and stability are mediated by the heavy chains of IαI and versican, promote leukocyte adhesion and accumulation by interacting with cell surface CD44 [72,73,74].

#### 4.3.1. Synovial Fluid and Arthritis

Synovial fluid functions as a lubricant in the cavity of synovial joints. HMW HA has been regarded as a primary lubricant molecule in synovial fluid due to its viscoelastic properties [75]. The concentration and molecular weight distribution of HA in synovial fluid vary with adult age and disease severity. Under inflammation, the degradation of synovial fluid HMW HA is accompanied by a loss of lubricating properties. Band et al. demonstrated that the molecular size of HA in the synovial fluid of inflamed joints was lower than in normal synovial fluid, suggesting that low molecular weight (LMW) HA arose from the depolymerization of HMW HA [76]. Osteoarthritis (OA) is a degenerative whole joint disease that has traditionally been classified as a non-inflammatory arthritis. However, it is now known that systemic inflammation is strongly implicated in OA pathogenesis. Shinozawa et al. revealed significantly higher CEMIP expression in the OA synovium [77]. The expression of CEMIP in OA synovial fibroblasts was strongly correlated with the distribution of LMW HA of less than 10^6^ Da in synovial fluid. Meanwhile, rheumatoid arthritis (RA) is a systemic autoimmune disease that causes chronic joint inflammation. Yoshida et al. investigated the expression patterns of HAS and HYAL genes in OA and RA synovium samples and found HYAL2 gene expression to be significantly higher in both types of arthritic synovia than in controls. Regarding HAS gene profiles, HAS3 gene expression in the RA synovium was higher than in the control synovium, with no obvious differences observed for the OA synovium [78]. To investigate whether the inhibition of HA synthesis exerted any inhibitory effects on arthritic inflammation, Yoshioka et al. used 4-MU in a mouse model of collagen-induced arthritis and found that the treatment dramatically decreased arthritis severity [79]. Furthermore, they observed that the triple knockdown of Has1, Has2, and Has3 in rheumatoid synovial fibroblasts exerted inhibitory effects on the expression of matrix metalloproteases, which played a critical role in cartilage destruction in RA joints. The above studies collectively demonstrate the significance of HA metabolism on the arthritic processes.

#### 4.3.2. Atherosclerosis

Atherosclerosis is a disease of chronic inflammation that is characterized by arterial narrowing and subsequent vascular compromise. Inflammatory and immune cells recruited to atherosclerotic lesions play critical roles in atherosclerosis initiation and progression. As a prominent component of atherosclerotic plaque ECM, HA stimulates the proliferation and migration of vascular smooth muscle cells (SMC) [80,81]. Activated vascular SMC manufacture large amounts of HA to form cable-like structures, which are necessary for inflammatory cell recruitment [71]. To investigate the pathological significance of HA accumulation in arterial walls, Chai et al. created transgenic mice overexpressing Has2 in SMCs and found that apolipoprotein E (ApoE) deficient mice with increased HA levels in vessel walls displayed accelerated atherosclerosis [82]. Moreover, a recent study using Has3/ApoE double deficient mice clearly demonstrated that Has3-mediated HA synthesis was critical in the development of atherosclerosis [83]. Absence of the Has3 gene reduced plaque inflammation as evidenced by fewer monocytes/macrophages and neutrophils in early atherosclerotic lesions [83]. Nieuwdorp et al. also reported that the perturbation of HA metabolism might be associated with the increased prevalence of atherosclerosis in type 1 diabetes patients [84]. Such studies implicate changes in HA metabolism as potential mechanisms involved in accelerated atherogenesis.

#### 4.3.3. Obesity and Metabolic Disorder

Obesity is a metabolic disorder characterized by excessive fat accumulation and chronic low-grade inflammation. A recent pharmacological study demonstrated that the inhibition of HA synthesis by 4-MU improved glucose metabolism and reduced body weight gain by activating BAT in mice on diabetogenic diet [31]. Possessing a great capacity for glucose and triglyceride uptake, BAT is an important tissue that maintains optimal thermogenesis and contributes to total body energy expenditure. Grandoch et al. investigated whether decreased HA synthesis interfered with glucose metabolism and adipose tissue function, and found that 4-MU treatment increased glycolysis, respiration and mitochondrial uncoupling protein 1 expression in BAT [31]. They also detected reduced adipose tissue mass coinciding with reduced inflammation following 4-MU treatment. Mice with double-knockout of Has2 and Has3, the most abundant HAS isoforms in BAT, partially mimicked the metabolic phenotypes of 4-MU-treated mice, suggesting that the inhibition of HA synthesis improved glucose metabolism by shifting glucose flux to glycolysis, possibly supporting mitochondrial respiration in BAT.

### 4.4. HA Metabolism and Cancer

Increased HA levels are closely associated with poor prognosis and survival in malignant cancers [85,86,87,88,89]. HAS expression in both the tumor and stroma of breast cancer has also been shown to relate to tumor aggressiveness and poor prognosis [90]. Emerging evidence has demonstrated a central role of HA in many aspects of tumor development and progression, such as cell proliferation, migration, invasion, angiogenesis, immune escape, and cancer stemness [16]. Elevated HA production by the forced expression of HAS isoforms enhanced tumor growth and metastasis in xenograft cancer models [91,92,93]. On the other hand, the knockdown of HAS2 in breast cancer cells suppressed their invasive capacity, which was rescued by HAS2 overexpression [94]. Contradictory observations have also been made in clinical and experimental studies. Reduced levels of tumor HA were associated with poor survival in squamous cell carcinoma patients [95]. Bharadwaj et al. demonstrated that the forced expression of HAS3 in prostate tumor cells suppressed tumorigenesis by retarding intrinsic growth [96]. This slow growth in cultures was restored either by exogenous addition of hyaluronidase or stable HYAL1 expression. The above findings underscore the importance of both HA biosynthesis and degradation for tumor cell growth.

LMW HA accumulation is associated with tumor aggressiveness in that it triggers the expression of specific cytokines and proteases required for TME remodeling [32,97,98]. Hyaluronidase-mediated HA degradation is the primary source of tumor-derived LMW HA. High levels of hyaluronidase activity were observed in human brain metastases [99] and metastatic breast cancer [100]. The expression of HYAL1 and HYAL2 was also elevated in melanoma [101], bladder cancer [102,103], and prostate cancer [104]. Moreover, CEMIP is overexpressed in many types of cancers with poor prognoses [105,106,107,108,109]. The knockdown of CEMIP or TMEM2 in breast cancer cells reduced cell motility and metastasis [110,111,112]. Meanwhile, decreased expression of HYAL1 and HYAL2 was associated with an unfavorable prognosis in ovarian [113], endometrial [114], and pancreatic cancers [115]. Indeed, the roles of HYAL1 and HYAL2 in cancer progression may vary depending on the cancer type.

Cancer cells are considered to remodel the TME to a unique set of conditions in which both HMW and LMW HA coexist by significantly enhancing the machinery involved in HA biosynthesis and degradation. HMW HA provides pericellular ECM that favors cancer cell proliferation and migration in addition to the intratumoral recruitment of such stromal cells as tumor-associated fibroblasts and macrophages. On the other hand, tumor-derived HA fragments promote angiogenic and immunosuppressive actions, either directly or through macrophage activation, and play an important role in cancer progression [18]. During tumor progression, the formation of new blood vessels supports the demand of oxygen and nutrients for tumor growth and ensuing metastatic processes. Tumor-associated macrophages (TAMs) are the major stromal cells in the TME [116] and secrete a panel of pro-angiogenic growth factors and cytokines including vascular endothelial growth factor, fibroblast growth factor-2, placental growth factor, and platelet-derived growth factor. Once macrophages infiltrate and immobilize into the tumor, they become polarized towards pro-angiogenic and pro-tumor M2-like phenotypes in response to TME components. Macrophages can be classified into two subtypes, M1 and M2, based on their surface marker expression and functional states [19]. M1 macrophages induce adaptive immune responses against cancer cells, while M2 macrophages mainly secrete the immunosuppressive cytokines interleukin-10 and transforming growth factor-β, and contribute to immunosuppression [117]. Several reports have suggested that tumor-derived HA fragments serve as a signal for macrophage polarization towards the M2 subtype [118,119], thereby contributing to tumor angiogenesis and the escape of cancer cells from immunosurveillance.

## 5. Metabolic Reprogramming Coupled with HA Production

**Figure 5:**
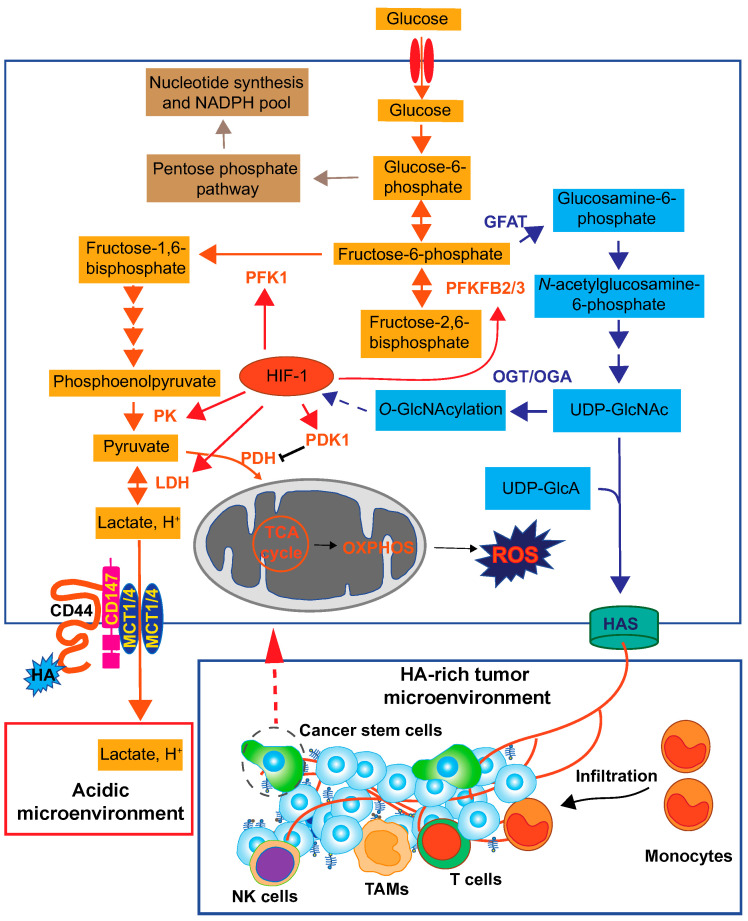
Overall view of HA roles in the cancer metabolic pathway. Cancer cells exhibit a high rate of glucose consumption for intracellular energy metabolism and macromolecule biosynthesis. Elevated HA biosynthesis accelerates the HBP along with a metabolic shift towards glycolysis. The enhanced HBP flux subsequently activates HIF-1 signaling, which is responsible for glycolytic enhancement. HIF-1 regulates the expression of key glycolytic enzymes such as pyruvate kinase (PK), 6-phosphofructo-1-kinase (PFK-1), 6-phosphofructo-2-kinase/fructose-2,6-bisphosphatase 2 and 3 (PFKFB2/3), pyruvate dehydrogenase kinase 1 (PDK1), and LDH [133]. As a consequence of the Warburg effect, the lactate is exported to the extracellular space via MCT1 and MCT4 and generates an acidic tumor microenvironment. HA-CD44 interactions promote the localization of EMMPRIN (CD147), MCT1, and MCT4 in the plasma membrane to efflux lactate. HA forms a HA-rich TME to facilitate monocyte/macrophage infiltration and cancer progression. OGA; *O*-GlcNAcase, OGT; *O*-GlcNAc transferase, OXPHOS; oxidative phosphorylation, PDH; pyruvate dehydrogenase.

Metabolic reprogramming has emerged as a critical determinant of malignant cells [120,121]. In order to sustain the demand of growth, proliferation, migration, and metastasis, cancer cells shift their metabolic phenotype by elevating glycolysis [122], inhibiting mitochondrial oxidative phosphorylation [123], and enhancing macromolecule biosynthesis [124] (Figure 5). The Warburg effect, or aerobic glycolysis, is a persistent cancer metabolism that adapts to rapid fluctuations in energy demand [122]. Upon the metabolic shift towards aerobic glycolysis, the high utilization of glucose by cancer cells results in the accumulation of an acidic product, lactate, in the TME (Figure 5). An acidic TME contributes to tumor progression by modulating cell migration, neovascularization, and tumor immune escape [121,125]. Metabolic switching is influenced by both cell-intrinsic and cell-extrinsic factors in the TME, one of which is the presence of ECM molecules such as HA [17]. We previously showed that HA overproduction in cancer cells activated the aerobic glycolysis pathway to influence growth, survival, and stemness [20]. Due to the upregulation of lactate dehydrogenase (LDH), HA-overproducing cancer cells secreted a large amount of lactate, resulting in an acidic TME. A xenograft model using Has3-overexpressing cancer cells also demonstrated HA accumulation in tumors to correlate with hypoxia and low tumor pH [126]. Proton-coupled monocarboxylate transporters (MCTs) are critical for lactate export across the plasma membrane [127]. Interestingly, the function of MCTs on lactate export in cancer may be modulated by HA signaling. HA-CD44 interactions promoted the localization of EMMPRIN (CD147), MCT1, and MCT4 in the plasma membrane to efflux lactate. Competitive inhibition of this signaling by HA oligosaccharides decreased the plasma membrane expression of CD44, MCT1, and MCT4 and reduced lactate efflux, thus confirming the role of HA and CD44 in lactate export [128]. Similarly, CD44v3-10 was found to co-express with EMMPRIN, multiple drug resistance 1 (MDR1), MCT1, and MCT4 in prostate cancer cells and associate with drug resistance and tumor progression [129]. The above observations suggest that HA may be involved in an acidic TME formation by modulating both glycolytic activation and lactate transport.

Since the excess synthesis of HA in cancer cells consumes large quantities of the UDP-GlcNAc and UDP-GlcA nucleotide sugar precursors, cancer cells need to accelerate the metabolic flux of nucleotide sugar synthesis to constantly maintain their cellular levels. Studies have shown that cancer cells adapt to active nucleotide sugar consumption by accelerating glucose uptake and HBP flux. Upregulation of glutamine: fructose-6-phosphate amidotransferase (GFAT), the rate limiting enzyme of HBP, and UDP-GlcNAc pyrophosphorylase 1 (UAP1), the final enzyme in the HBP, was observed in human breast and prostate cancers, respectively [130,131]. In breast cancer, elevated GFAT expression was in parallel with an increase in UDP-GlcNAc content and was strongly correlated with tumor HA levels [131]. This was supported by our recent finding that GFAT and HAS2 were co-expressed in malignant breast cancer cells [132]. We furthermore identified a metabolomic signature characteristic of HA-overproducing breast cancer cells [20]. Even though up to 2–5% of glucose enters the HBP under normal conditions, a massive flow of glucose metabolites to the HBP has been observed in HA-overproducing cancer cells. These observations suggest the existence of a positive feedback loop between the HBP and HA production. Moreover, metabolomic analyses detected significant amounts of glycolytic metabolites in HA-overproducing breast cancer cells [20]. Pharmacological inhibition and gene silencing of GFAT have revealed HA-driven glycolytic enhancement to be regulated by hypoxia-inducible factor (HIF-1) signaling under the control of HBP flux. HIF-1, a hetero dimer composed of α and β subunits, functions as a central transcription factor that regulates the gene expression of many glycolytic enzymes [133]. Of note, the stability of the HIF-1α subunit seems to be promoted by a mechanism involving enhanced HBP flux and *O*-GlcNAcylation. Ferrer et al. provided direct evidence that the HBP modulated metabolic reprogramming in cancer cells by regulating HIF-1α stabilization via *O*-GlcNAcylation [134]. Thus, increased HBP flux and subsequent *O*-GlcNAcylation may be responsible for HA-driven glycolytic enhancement. Unlike differentiated cancer cells, CSCs preferentially rely on glycolytic pathways. Compared with non-CSCs, enhanced glucose consumption, lactate production, and ATP levels were observed in CSCs [135,136,137]. We recently discovered that HA production promoted CSC-like properties, such as mammosphere formation and tumorigenic abilities, via the metabolic reprogramming of glycolysis and the HBP [20]. Given these findings, HA-induced metabolic reprogramming may be a central event in CSC regulation.

## 6. Conclusions

This review summarizes the current evidence on the close relationship between HA metabolism and functions. Genetic engineering and pharmacological approaches to artificially alter HA metabolism have provided new insights into the functions of HA in tissue morphogenesis and homeostasis. Furthermore, recent metabolomic approaches have demonstrated novel HA roles for modulating cellular behaviors via the reprogramming of cellular metabolism coupled to HA production. In some events during morphogenesis and in certain diseases including cancer, HA biosynthesis and degradation are significantly enhanced, and its turnover rate may be accelerated in such states. Therefore, the balance between HA synthesis and degradation not only regulates cellular functions by controlling the concentration and molecular size of extracellular HA, but may also modulate intracellular metabolism by controlling HA turnover. A more complete understanding of the relationship between HA metabolism and functions will assist in the elucidation of the multifaceted roles of HA and possibly contribute to overcoming various diseases suspectedly caused by abnormal HA metabolism.

## References

[1] Meyer K., Palmer J.W. (1934). The polysaccharide of the vitreous humor. J. Biol. Chem..

[2] Weissmann B., Meyer K. (1954). The structure of hyalobiuronic acid and of hyaluronic acid from umbilical cord1,2. J. Am. Chem. Soc..

[3] Holmes M.W., Bayliss M.T., Muir H. (1988). Hyaluronic acid in human articular cartilage. Age-related changes in content and size. Biochem. J..

[4] Cowman M.K., Lee H.G., Schwertfeger K.L., McCarthy J.B., Turley E.A. (2015). The content and size of hyaluronan in biological fluids and tissues. Front. Immunol..

[5] Hascall V., Esko J.D., Varki A., Cummings R.D., Esko J.D., Stanley P., Hart G.W., Aebi M., Darvill A.G., Kinoshita T., Packer N.H., Prestegard J.H. (2015). Hyaluronan. Essentials of Glycobiology.

[6] Itano N., Sawai T., Yoshida M., Lenas P., Yamada Y., Imagawa M., Shinomura T., Hamaguchi M., Yoshida Y., Ohnuki Y. (1999). Three isoforms of mammalian hyaluronan synthases have distinct enzymatic properties. J. Biol. Chem..

[7] Csoka A.B., Frost G.I., Stern R. (2001). The six hyaluronidase-like genes in the human and mouse genomes. Matrix Biol..

[8] Yoshida H., Nagaoka A., Kusaka-Kikushima A., Tobiishi M., Kawabata K., Sayo T., Sakai S., Sugiyama Y., Enomoto H., Okada Y. (2013). KIAA1199, a deafness gene of unknown function, is a new hyaluronan binding protein involved in hyaluronan depolymerization. Proc. Natl. Acad. Sci. USA.

[9] Yamamoto H., Tobisawa Y., Inubushi T., Irie F., Ohyama C., Yamaguchi Y. (2017). A mammalian homolog of the zebrafish transmembrane protein 2 (TMEM2) is the long-sought-after cell-surface hyaluronidase. J. Biol. Chem..

[10] Gribbon P., Heng B.C., Hardingham T.E. (2000). The analysis of intermolecular interactions in concentrated hyaluronan solutions suggest no evidence for chain-chain association. Biochem. J..

[11] Toole B.P. (1990). Hyaluronan and its binding proteins, the hyaladherins. Curr. Opin. Cell Biol..

[12] Hascall V.C., Majors A.K., De La Motte C.A., Evanko S.P., Wang A., Drazba J.A., Strong S.A., Wight T.N. (2004). Intracellular hyaluronan: A new frontier for inflammation?. Biochim. Biophys. Acta.

[13] Tighe R.M., Garantziotis S. (2019). Hyaluronan interactions with innate immunity in lung biology. Matrix Biol..

[14] Tavianatou A.G., Caon I., Franchi M., Piperigkou Z., Galesso D., Karamanos N.K. (2019). Hyaluronan: Molecular size-dependent signaling and biological functions in inflammation and cancer. FEBS J..

[15] Misra S., Hascall V.C., Markwald R.R., Ghatak S. (2015). Interactions between hyaluronan and its receptors (CD44, RHAMM) regulate the activities of inflammation and cancer. Front. Immunol..

[16] Toole B.P. (2004). Hyaluronan: From extracellular glue to pericellular cue. Nat. Rev. Cancer.

[17] Chanmee T., Ontong P., Itano N. (2016). Hyaluronan: A modulator of the tumor microenvironment. Cancer Lett..

[18] West D.C., Kumar S. (1989). Hyaluronan and angiogenesis. Ciba Found. Symp..

[19] Pathria P., Louis T.L., Varner J.A. (2019). Targeting tumor-associated macrophages in cancer. Trends Immunol..

[20] Chanmee T., Ontong P., Izumikawa T., Higashide M., Mochizuki N., Chokchaitaweesuk C., Khansai M., Nakajima K., Kakizaki I., Kongtawelert P. (2016). Hyaluronan production regulates metabolic and cancer stem-like properties of breast cancer cells via hexosamine biosynthetic pathway-coupled HIF-1 signaling. J. Biol. Chem..

[21] Terabe K., Ohashi Y., Tsuchiya S., Ishizuka S., Knudson C.B., Knudson W. (2019). Chondroprotective effects of 4-methylumbelliferone and hyaluronan synthase-2 overexpression involve changes in chondrocyte energy metabolism. J. Biol. Chem..

[22] Itano N., Kimata K. (2002). Mammalian hyaluronan synthases. IUBMB Life.

[23] Weigel P.H. (2015). Hyaluronan synthase: The mechanism of initiation at the reducing end and a pendulum model for polysaccharide translocation to the cell exterior. Int. J. Cell Biol..

[24] Goentzel B.J., Weigel P.H., Steinberg R.A. (2006). Recombinant human hyaluronan synthase 3 is phosphorylated in mammalian cells. Biochem. J..

[25] Vigetti D., Clerici M., Deleonibus S., Karousou E., Viola M., Moretto P., Heldin P., Hascall V.C., De Luca G., Passi A. (2011). Hyaluronan synthesis is inhibited by adenosine monophosphate-activated protein kinase through the regulation of HAS2 activity in human aortic smooth muscle cells. J. Biol. Chem..

[26] Vigetti D., Deleonibus S., Moretto P., Karousou E., Viola M., Bartolini B., Hascall V.C., Tammi M., De Luca G., Passi A. (2012). Role of UDP-N-acetylglucosamine (GlcNAc) and O-GlcNAcylation of hyaluronan synthase 2 in the control of chondroitin sulfate and hyaluronan synthesis. J. Biol. Chem..

[27] Karousou E., Kamiryo M., Skandalis S.S., Ruusala A., Asteriou T., Passi A., Yamashita H., Hellman U., Heldin C.H., Heldin P. (2010). The activity of hyaluronan synthase 2 is regulated by dimerization and ubiquitination. J. Biol. Chem..

[28] Vigetti D., Deleonibus S., Moretto P., Bowen T., Fischer J.W., Grandoch M., Oberhuber A., Love D.C., Hanover J.A., Cinquetti R. (2014). Natural antisense transcript for hyaluronan synthase 2 (HAS2-AS1) induces transcription of HAS2 via protein O-GlcNAcylation. J. Biol. Chem..

[29] Kakizaki I., Kojima K., Takagaki K., Endo M., Kannagi R., Ito M., Maruo Y., Sato H., Yasuda T., Mita S. (2004). A novel mechanism for the inhibition of hyaluronan biosynthesis by 4-methylumbelliferone. J. Biol. Chem..

[30] Jokela T.A., Makkonen K.M., Oikari S., Karna R., Koli E., Hart G.W., Tammi R.H., Carlberg C., Tammi M.I. (2011). Cellular content of UDP-N-acetylhexosamines controls hyaluronan synthase 2 expression and correlates with O-linked N-acetylglucosamine modification of transcription factors YY1 and SP1. J. Biol. Chem..

[31] Grandoch M., Flogel U., Virtue S., Maier J.K., Jelenik T., Kohlmorgen C., Feldmann K., Ostendorf Y., Castaneda T.R., Zhou Z. (2019). 4-Methylumbelliferone improves the thermogenic capacity of brown adipose tissue. Nat. Metab..

[32] Stern R., Asari A.A., Sugahara K.N. (2006). Hyaluronan fragments: An information-rich system. Eur. J. Cell Biol..

[33] Harada H., Takahashi M. (2007). CD44-dependent intracellular and extracellular catabolism of hyaluronic acid by hyaluronidase-1 and -2. J. Biol. Chem..

[34] Atmuri V., Martin D.C., Hemming R., Gutsol A., Byers S., Sahebjam S., Thliveris J.A., Mort J.S., Carmona E., Anderson J.E. (2008). Hyaluronidase 3 (HYAL3) knockout mice do not display evidence of hyaluronan accumulation. Matrix Biol..

[35] Kaneiwa T., Mizumoto S., Sugahara K., Yamada S. (2010). Identification of human hyaluronidase-4 as a novel chondroitin sulfate hydrolase that preferentially cleaves the galactosaminidic linkage in the trisulfated tetrasaccharide sequence. Glycobiology.

[36] Martin-Deleon P.A. (2011). Germ-cell hyaluronidases: Their roles in sperm function. Int. J. Androl..

[37] Culty M., Nguyen H.A., Underhill C.B. (1992). The hyaluronan receptor (CD44) participates in the uptake and degradation of hyaluronan. J. Cell Biol..

[38] Hua Q., Knudson C.B., Knudson W. (1993). Internalization of hyaluronan by chondrocytes occurs via receptor-mediated endocytosis. J. Cell Sci..

[39] Afify A.M., Stern M., Guntenhoner M., Stern R. (1993). Purification and characterization of human serum hyaluronidase. Arch. Biochem. Biophys..

[40] Rai S.K., Duh F.M., Vigdorovich V., Danilkovitch-Miagkova A., Lerman M.I., Miller A.D. (2001). Candidate tumor suppressor HYAL2 is a glycosylphosphatidylinositol (GPI)-anchored cell-surface receptor for jaagsiekte sheep retrovirus, the envelope protein of which mediates oncogenic transformation. Proc. Natl. Acad. Sci. USA.

[41] Jadin L., Wu X., Ding H., Frost G.I., Onclinx C., Triggs-Raine B., Flamion B. (2008). Skeletal and hematological anomalies in HYAL2-deficient mice: A second type of mucopolysaccharidosis IX?. FASEB J..

[42] Fraser J.R., Laurent T.C., Pertoft H., Baxter E. (1981). Plasma clearance, tissue distribution and metabolism of hyaluronic acid injected intravenously in the rabbit. Biochem. J..

[43] Fraser J.R., Appelgren L.E., Laurent T.C. (1983). Tissue uptake of circulating hyaluronic acid. A whole body autoradiographic study. Cell Tissue Res..

[44] Zhou B., Oka J.A., Singh A., Weigel P.H. (1999). Purification and subunit characterization of the rat liver endocytic hyaluronan receptor. J. Biol. Chem..

[45] McGary C.T., Raja R.H., Weigel P.H. (1989). Endocytosis of hyaluronic acid by rat liver endothelial cells. Evidence for receptor recycling. Biochem. J..

[46] McGary C.T., Yannariello-Brown J., Kim D.W., Stinson T.C., Weigel P.H. (1993). Degradation and intracellular accumulation of a residualizing hyaluronan derivative by liver endothelial cells. Hepatology.

[47] Scott J.E., Cummings C., Brass A., Chen Y. (1991). Secondary and tertiary structures of hyaluronan in aqueous solution, investigated by rotary shadowing-electron microscopy and computer simulation. Hyaluronan is a very efficient network-forming polymer. Biochem. J..

[48] Laurent T.C., Laurent U.B., Fraser J.R. (1996). The structure and function of hyaluronan: An overview. Immunol. Cell Biol..

[49] Cowman M.K., Schmidt T.A., Raghavan P., Stecco A. (2015). Viscoelastic properties of hyaluronan in physiological conditions. F1000Res.

[50] Comper W.D., Laurent T.C. (1978). Physiological function of connective tissue polysaccharides. Physiol. Rev..

[51] Gribbon P., Heng B.C., Hardingham T.E. (1999). The molecular basis of the solution properties of hyaluronan investigated by confocal fluorescence recovery after photobleaching. Biophys. J..

[52] Slevin M., Kumar S., Gaffney J. (2002). Angiogenic oligosaccharides of hyaluronan induce multiple signaling pathways affecting vascular endothelial cell mitogenic and wound healing responses. J. Biol. Chem..

[53] Jiang D., Liang J., Noble P.W. (2011). Hyaluronan as an immune regulator in human diseases. Physiol. Rev..

[54] Campo G.M., Avenoso A., Campo S., D’Ascola A., Nastasi G., Calatroni A. (2010). Small hyaluronan oligosaccharides induce inflammation by engaging both toll-like-4 and CD44 receptors in human chondrocytes. Biochem. Pharmacol..

[55] Cowman M.K., Spagnoli C., Kudasheva D., Li M., Dyal A., Kanai S., Balazs E.A. (2005). Extended, relaxed, and condensed conformations of hyaluronan observed by atomic force microscopy. Biophys. J..

[56] Toole B.P. (2001). Hyaluronan in morphogenesis. Semin. Cell Dev. Biol..

[57] Camenisch T.D., Spicer A.P., Brehm-Gibson T., Biesterfeldt J., Augustine M.L., Calabro A., Kubalak S., Klewer S.E., McDonald J.A. (2000). Disruption of hyaluronan synthase-2 abrogates normal cardiac morphogenesis and hyaluronan-mediated transformation of epithelium to mesenchyme. J. Clin. Investig..

[58] Camenisch T.D., Schroeder J.A., Bradley J., Klewer S.E., McDonald J.A. (2002). Heart-valve mesenchyme formation is dependent on hyaluronan-augmented activation of ErbB2-ErbB3 receptors. Nat. Med..

[59] Chowdhury B., Hemming R., Hombach-Klonisch S., Flamion B., Triggs-Raine B. (2013). Murine hyaluronidase 2 deficiency results in extracellular hyaluronan accumulation and severe cardiopulmonary dysfunction. J. Biol. Chem..

[60] Chowdhury B., Xiang B., Muggenthaler M., Dolinsky V.W., Triggs-Raine B. (2016). Hyaluronidase 2 deficiency is a molecular cause of cor triatriatum sinister in mice. Int. J. Cardiol..

[61] Chowdhury B., Xiang B., Liu M., Hemming R., Dolinsky V.W., Triggs-Raine B. (2017). Hyaluronidase 2 deficiency causes increased mesenchymal cells, congenital heart defects, and heart failure. Circ. Cardiovasc. Genet..

[62] Matsumoto K., Li Y., Jakuba C., Sugiyama Y., Sayo T., Okuno M., Dealy C.N., Toole B.P., Takeda J., Yamaguchi Y. (2009). Conditional inactivation of Has2 reveals a crucial role for hyaluronan in skeletal growth, patterning, chondrocyte maturation and joint formation in the developing limb. Development.

[63] Huang Y., Askew E.B., Knudson C.B., Knudson W. (2016). CRISPR/Cas9 knockout of HAS2 in rat chondrosarcoma chondrocytes demonstrates the requirement of hyaluronan for aggrecan retention. Matrix Biol..

[64] Shimoda M., Yoshida H., Mizuno S., Hirozane T., Horiuchi K., Yoshino Y., Hara H., Kanai Y., Inoue S., Ishijima M. (2017). Hyaluronan-binding protein involved in hyaluronan depolymerization controls endochondral ossification through hyaluronan metabolism. Am. J. Pathol..

[65] Sivakumar A., Mahadevan A., Lauer M.E., Narvaez R.J., Ramesh S., Demler C.M., Souchet N.R., Hascall V.C., Midura R.J., Garantziotis S. (2018). Midgut laterality is driven by hyaluronan on the right. Dev. Cell.

[66] Aya K.L., Stern R. (2014). Hyaluronan in wound healing: Rediscovering a major player. Wound Repair Regen..

[67] Mack J.A., Feldman R.J., Itano N., Kimata K., Lauer M., Hascall V.C., Maytin E.V. (2012). Enhanced inflammation and accelerated wound closure following tetraphorbol ester application or full-thickness wounding in mice lacking hyaluronan synthases Has1 and Has3. J. Investig. Dermatol..

[68] Fronza M., Caetano G.F., Leite M.N., Bitencourt C.S., Paula-Silva F.W., Andrade T.A., Frade M.A., Merfort I., Faccioli L.H. (2014). Hyaluronidase modulates inflammatory response and accelerates the cutaneous wound healing. PLoS ONE.

[69] Buhren B., Schrumpf H., Gorges K., Reiners O., Boelke E., Fischer J., Homey B., Gerber A. (2020). Dose- and time-dependent effects of hyaluronidase on structural cells and the extracellular matrix of the skin. Eur. J. Med. Res..

[70] Dong Y., Arif A., Olsson M., Cali V., Hardman B., Dosanjh M., Lauer M., Midura R.J., Hascall V.C., Brown K.L. (2016). Endotoxin free hyaluronan and hyaluronan fragments do not stimulate TNF-alpha, interleukin-12 or upregulate co-stimulatory molecules in dendritic cells or macrophages. Sci. Rep..

[71] Petrey A.C., de la Motte C.A. (2014). Hyaluronan, a crucial regulator of inflammation. Front. Immunol..

[72] de la Motte C.A., Hascall V.C., Drazba J., Bandyopadhyay S.K., Strong S.A. (2003). Mononuclear leukocytes bind to specific hyaluronan structures on colon mucosal smooth muscle cells treated with polyinosinic acid:polycytidylic acid: Inter-alpha-trypsin inhibitor is crucial to structure and function. Am. J. Pathol..

[73] Kang I., Harten I.A., Chang M.Y., Braun K.R., Sheih A., Nivison M.P., Johnson P.Y., Workman G., Kaber G., Evanko S.P. (2017). Versican Deficiency Significantly Reduces Lung Inflammatory Response Induced by Polyinosine-Polycytidylic Acid Stimulation. J. Biol. Chem..

[74] Day A.J., de la Motte C.A. (2005). Hyaluronan cross-linking: A protective mechanism in inflammation?. Trends Immunol..

[75] Tamer T.M. (2013). Hyaluronan and synovial joint: Function, distribution and healing. Interdiscip. Toxicol..

[76] Band P.A., Heeter J., Wisniewski H.G., Liublinska V., Pattanayak C.W., Karia R.J., Stabler T., Balazs E.A., Kraus V.B. (2015). Hyaluronan molecular weight distribution is associated with the risk of knee osteoarthritis progression. Osteoarthr. Cartil..

[77] Shiozawa J., de Vega S., Cilek M.Z., Yoshinaga C., Nakamura T., Kasamatsu S., Yoshida H., Kaneko H., Ishijima M., Kaneko K. (2020). Implication of HYBID (Hyaluronan-binding protein involved in hyaluronan depolymerization) in hyaluronan degradation by synovial fibroblasts in patients with knee osteoarthritis. Am. J. Pathol..

[78] Yoshida M., Sai S., Marumo K., Tanaka T., Itano N., Kimata K., Fujii K. (2004). Expression analysis of three isoforms of hyaluronan synthase and hyaluronidase in the synovium of knees in osteoarthritis and rheumatoid arthritis by quantitative real-time reverse transcriptase polymerase chain reaction. Arthritis Res. Ther..

[79] Yoshioka Y., Kozawa E., Urakawa H., Arai E., Futamura N., Zhuo L., Kimata K., Ishiguro N., Nishida Y. (2013). Suppression of hyaluronan synthesis alleviates inflammatory responses in murine arthritis and in human rheumatoid synovial fibroblasts. Arthritis Rheum..

[80] Papakonstantinou E., Roth M., Block L.H., Mirtsou-Fidani V., Argiriadis P., Karakiulakis G. (1998). The differential distribution of hyaluronic acid in the layers of human atheromatic aortas is associated with vascular smooth muscle cell proliferation and migration. Atherosclerosis.

[81] Sussmann M., Sarbia M., Meyer-Kirchrath J., Nusing R.M., Schror K., Fischer J.W. (2004). Induction of hyaluronic acid synthase 2 (HAS2) in human vascular smooth muscle cells by vasodilatory prostaglandins. Circ. Res..

[82] Chai S., Chai Q., Danielsen C.C., Hjorth P., Nyengaard J.R., Ledet T., Yamaguchi Y., Rasmussen L.M., Wogensen L. (2005). Overexpression of hyaluronan in the tunica media promotes the development of atherosclerosis. Circ. Res..

[83] Homann S., Grandoch M., Kiene L.S., Podsvyadek Y., Feldmann K., Rabausch B., Nagy N., Lehr S., Kretschmer I., Oberhuber A. (2018). Hyaluronan synthase 3 promotes plaque inflammation and atheroprogression. Matrix Biol..

[84] Nieuwdorp M., Holleman F., de Groot E., Vink H., Gort J., Kontush A., Chapman M.J., Hutten B.A., Brouwer C.B., Hoekstra J.B. (2007). Perturbation of hyaluronan metabolism predisposes patients with type 1 diabetes mellitus to atherosclerosis. Diabetologia.

[85] Auvinen P., Tammi R., Parkkinen J., Tammi M., Agren U., Johansson R., Hirvikoski P., Eskelinen M., Kosma V.M. (2000). Hyaluronan in peritumoral stroma and malignant cells associates with breast cancer spreading and predicts survival. Am. J. Pathol..

[86] Josefsson A., Adamo H., Hammarsten P., Granfors T., Stattin P., Egevad L., Laurent A.E., Wikstrom P., Bergh A. (2011). Prostate cancer increases hyaluronan in surrounding nonmalignant stroma, and this response is associated with tumor growth and an unfavorable outcome. Am. J. Pathol..

[87] Lipponen P., Aaltomaa S., Tammi R., Tammi M., Agren U., Kosma V.M. (2001). High stromal hyaluronan level is associated with poor differentiation and metastasis in prostate cancer. Eur. J. Cancer.

[88] Anttila M.A., Tammi R.H., Tammi M.I., Syrjanen K.J., Saarikoski S.V., Kosma V.M. (2000). High levels of stromal hyaluronan predict poor disease outcome in epithelial ovarian cancer. Cancer Res..

[89] Pirinen R., Tammi R., Tammi M., Hirvikoski P., Parkkinen J.J., Johansson R., Bohm J., Hollmen S., Kosma V.M. (2001). Prognostic value of hyaluronan expression in non-small-cell lung cancer: Increased stromal expression indicates unfavorable outcome in patients with adenocarcinoma. Int. J. Cancer.

[90] Auvinen P., Rilla K., Tumelius R., Tammi M., Sironen R., Soini Y., Kosma V.M., Mannermaa A., Viikari J., Tammi R. (2014). Hyaluronan synthases (HAS1-3) in stromal and malignant cells correlate with breast cancer grade and predict patient survival. Breast Cancer Res. Treat..

[91] Itano N., Sawai T., Miyaishi O., Kimata K. (1999). Relationship between hyaluronan production and metastatic potential of mouse mammary carcinoma cells. Cancer Res..

[92] Liu N., Gao F., Han Z., Xu X., Underhill C.B., Zhang L. (2001). Hyaluronan synthase 3 overexpression promotes the growth of TSU prostate cancer cells. Cancer Res..

[93] Kosaki R., Watanabe K., Yamaguchi Y. (1999). Overproduction of hyaluronan by expression of the hyaluronan synthase Has2 enhances anchorage-independent growth and tumorigenicity. Cancer Res..

[94] Bernert B., Porsch H., Heldin P. (2011). Hyaluronan synthase 2 (HAS2) promotes breast cancer cell invasion by suppression of tissue metalloproteinase inhibitor 1 (TIMP-1). J. Biol. Chem..

[95] Kosunen A., Ropponen K., Kellokoski J., Pukkila M., Virtaniemi J., Valtonen H., Kumpulainen E., Johansson R., Tammi R., Tammi M. (2004). Reduced expression of hyaluronan is a strong indicator of poor survival in oral squamous cell carcinoma. Oral Oncol..

[96] Bharadwaj A.G., Rector K., Simpson M.A. (2007). Inducible hyaluronan production reveals differential effects on prostate tumor cell growth and tumor angiogenesis. J. Biol. Chem..

[97] Schmaus A., Klusmeier S., Rothley M., Dimmler A., Sipos B., Faller G., Thiele W., Allgayer H., Hohenberger P., Post S. (2014). Accumulation of small hyaluronan oligosaccharides in tumour interstitial fluid correlates with lymphatic invasion and lymph node metastasis. Br. J. Cancer.

[98] Sugahara K.N., Murai T., Nishinakamura H., Kawashima H., Saya H., Miyasaka M. (2003). Hyaluronan oligosaccharides induce CD44 cleavage and promote cell migration in CD44-expressing tumor cells. J. Biol. Chem..

[99] Delpech B., Laquerriere A., Maingonnat C., Bertrand P., Freger P. (2002). Hyaluronidase is more elevated in human brain metastases than in primary brain tumours. Anticancer Res..

[100] Victor R., Chauzy C., Girard N., Gioanni J., d’Anjou J., Stora De Novion H., Delpech B. (1999). Human breast-cancer metastasis formation in a nude-mouse model: Studies of hyaluronidase, hyaluronan and hyaluronan-binding sites in metastatic cells. Int. J. Cancer.

[101] Siiskonen H., Poukka M., Tyynela-Korhonen K., Sironen R., Pasonen-Seppanen S. (2013). Inverse expression of hyaluronidase 2 and hyaluronan synthases 1-3 is associated with reduced hyaluronan content in malignant cutaneous melanoma. BMC Cancer.

[102] Lokeshwar V.B., Young M.J., Goudarzi G., Iida N., Yudin A.I., Cherr G.N., Selzer M.G. (1999). Identification of bladder tumor-derived hyaluronidase: Its similarity to HYAL1. Cancer Res..

[103] Morera D.S., Hennig M.S., Talukder A., Lokeshwar S.D., Wang J., Garcia-Roig M., Ortiz N., Yates T.J., Lopez L.E., Kallifatidis G. (2017). Hyaluronic acid family in bladder cancer: Potential prognostic biomarkers and therapeutic targets. Br. J. Cancer.

[104] Posey J.T., Soloway M.S., Ekici S., Sofer M., Civantos F., Duncan R.C., Lokeshwar V.B. (2003). Evaluation of the prognostic potential of hyaluronic acid and hyaluronidase (HYAL1) for prostate cancer. Cancer Res..

[105] Kuscu C., Evensen N., Kim D., Hu Y.J., Zucker S., Cao J. (2012). Transcriptional and epigenetic regulation of KIAA1199 gene expression in human breast cancer. PLoS ONE.

[106] Koga A., Sato N., Kohi S., Yabuki K., Cheng X.B., Hisaoka M., Hirata K. (2017). KIAA1199/CEMIP/HYBID overexpression predicts poor prognosis in pancreatic ductal adenocarcinoma. Pancreatology.

[107] Deng F., Lei J., Zhang X., Huang W., Li Y., Wu D. (2017). Overexpression of KIAA1199: An independent prognostic marker in nonsmall cell lung cancer. J. Cancer Res. Ther..

[108] Fink S.P., Myeroff L.L., Kariv R., Platzer P., Xin B., Mikkola D., Lawrence E., Morris N., Nosrati A., Willson J.K. (2015). Induction of KIAA1199/CEMIP is associated with colon cancer phenotype and poor patient survival. Oncotarget.

[109] Jia S., Qu T., Wang X., Feng M., Yang Y., Feng X., Ma R., Li W., Hu Y., Feng Y. (2017). KIAA1199 promotes migration and invasion by Wnt/beta-catenin pathway and MMPs mediated EMT progression and serves as a poor prognosis marker in gastric cancer. PLoS ONE.

[110] Evensen N.A., Kuscu C., Nguyen H.L., Zarrabi K., Dufour A., Kadam P., Hu Y.J., Pulkoski-Gross A., Bahou W.F., Zucker S. (2013). Unraveling the role of KIAA1199, a novel endoplasmic reticulum protein, in cancer cell migration. J. Natl. Cancer Inst..

[111] Jami M.S., Hou J., Liu M., Varney M.L., Hassan H., Dong J., Geng L., Wang J., Yu F., Huang X. (2014). Functional proteomic analysis reveals the involvement of KIAA1199 in breast cancer growth, motility and invasiveness. BMC Cancer.

[112] Lee H., Goodarzi H., Tavazoie S.F., Alarcon C.R. (2016). TMEM2 Is a SOX4-regulated gene that mediates metastatic migration and invasion in breast cancer. Cancer Res..

[113] Nykopp T.K., Rilla K., Sironen R., Tammi M.I., Tammi R.H., Hamalainen K., Heikkinen A.M., Komulainen M., Kosma V.M., Anttila M. (2009). Expression of hyaluronan synthases (HAS1-3) and hyaluronidases (HYAL1-2) in serous ovarian carcinomas: Inverse correlation between HYAL1 and hyaluronan content. BMC Cancer.

[114] Nykopp T.K., Pasonen-Seppanen S., Tammi M.I., Tammi R.H., Kosma V.M., Anttila M., Sironen R. (2015). Decreased hyaluronidase 1 expression is associated with early disease recurrence in human endometrial cancer. Gynecol. Oncol..

[115] Cheng X.B., Sato N., Kohi S., Yamaguchi K. (2013). Prognostic impact of hyaluronan and its regulators in pancreatic ductal adenocarcinoma. PLoS ONE.

[116] Chanmee T., Ontong P., Konno K., Itano N. (2014). Tumor-associated macrophages as major players in the tumor microenvironment. Cancers.

[117] Li X., Liu R., Su X., Pan Y., Han X., Shao C., Shi Y. (2019). Harnessing tumor-associated macrophages as aids for cancer immunotherapy. Mol. Cancer.

[118] Zhang G., Guo L., Yang C., Liu Y., He Y., Du Y., Wang W., Gao F. (2016). A novel role of breast cancer-derived hyaluronan on inducement of M2-like tumor-associated macrophages formation. Oncoimmunology.

[119] Kuang D.M., Wu Y., Chen N., Cheng J., Zhuang S.M., Zheng L. (2007). Tumor-derived hyaluronan induces formation of immunosuppressive macrophages through transient early activation of monocytes. Blood.

[120] Pavlova N.N., Thompson C.B. (2016). The Emerging Hallmarks of Cancer Metabolism. Cell Metab..

[121] Leone R.D., Powell J.D. (2020). Metabolism of immune cells in cancer. Nat. Rev. Cancer.

[122] Liberti M.V., Locasale J.W. (2016). The warburg effect: How does it benefit cancer cells?. Trends Biochem. Sci..

[123] Shiratori R., Furuichi K., Yamaguchi M., Miyazaki N., Aoki H., Chibana H., Ito K., Aoki S. (2019). Glycolytic suppression dramatically changes the intracellular metabolic profile of multiple cancer cell lines in a mitochondrial metabolism-dependent manner. Sci. Rep..

[124] DeBerardinis R.J., Chandel N.S. (2016). Fundamentals of cancer metabolism. Sci Adv.

[125] Boedtkjer E., Pedersen S.F. (2020). The acidic tumor microenvironment as a driver of cancer. Annu. Rev. Physiol..

[126] Li X., Shepard H.M., Cowell J.A., Zhao C., Osgood R.J., Rosengren S., Blouw B., Garrovillo S.A., Pagel M.D., Whatcott C.J. (2018). Parallel accumulation of tumor hyaluronan, collagen, and other drivers of tumor progression. Clin. Cancer Res..

[127] Payen V.L., Mina E., Van Hee V.F., Porporato P.E., Sonveaux P. (2020). Monocarboxylate transporters in cancer. Mol Metab.

[128] Slomiany M.G., Grass G.D., Robertson A.D., Yang X.Y., Maria B.L., Beeson C., Toole B.P. (2009). Hyaluronan, CD44, and emmprin regulate lactate efflux and membrane localization of monocarboxylate transporters in human breast carcinoma cells. Cancer Res..

[129] Hao J., Chen H., Madigan M.C., Cozzi P.J., Beretov J., Xiao W., Delprado W.J., Russell P.J., Li Y. (2010). Co-expression of CD147 (EMMPRIN), CD44v3-10, MDR1 and monocarboxylate transporters is associated with prostate cancer drug resistance and progression. Br. J. Cancer.

[130] Itkonen H.M., Engedal N., Babaie E., Luhr M., Guldvik I.J., Minner S., Hohloch J., Tsourlakis M.C., Schlomm T., Mills I.G. (2015). UAP1 is overexpressed in prostate cancer and is protective against inhibitors of N-linked glycosylation. Oncogene.

[131] Oikari S., Kettunen T., Tiainen S., Hayrinen J., Masarwah A., Sudah M., Sutela A., Vanninen R., Tammi M., Auvinen P. (2018). UDP-sugar accumulation drives hyaluronan synthesis in breast cancer. Matrix Biol..

[132] Chokchaitaweesuk C., Kobayashi T., Izumikawa T., Itano N. (2019). Enhanced hexosamine metabolism drives metabolic and signaling networks involving hyaluronan production and O-GlcNAcylation to exacerbate breast cancer. Cell Death Dis..

[133] Ngo H., Tortorella S.M., Ververis K., Karagiannis T.C. (2015). The Warburg effect: Molecular aspects and therapeutic possibilities. Mol. Biol. Rep..

[134] Ferrer C.M., Lynch T.P., Sodi V.L., Falcone J.N., Schwab L.P., Peacock D.L., Vocadlo D.J., Seagroves T.N., Reginato M.J. (2014). O-GlcNAcylation regulates cancer metabolism and survival stress signaling via regulation of the HIF-1 pathway. Mol. Cell.

[135] Ciavardelli D., Rossi C., Barcaroli D., Volpe S., Consalvo A., Zucchelli M., De Cola A., Scavo E., Carollo R., D’Agostino D. (2014). Breast cancer stem cells rely on fermentative glycolysis and are sensitive to 2-deoxyglucose treatment. Cell Death Dis..

[136] Peng F., Wang J.H., Fan W.J., Meng Y.T., Li M.M., Li T.T., Cui B., Wang H.F., Zhao Y., An F. (2018). Glycolysis gatekeeper PDK1 reprograms breast cancer stem cells under hypoxia. Oncogene.

[137] Deshmukh A., Deshpande K., Arfuso F., Newsholme P., Dharmarajan A. (2016). Cancer stem cell metabolism: A potential target for cancer therapy. Mol. Cancer.

